# TRIM2 directly deubiquitinates and stabilizes Snail1 protein, mediating proliferation and metastasis of lung adenocarcinoma

**DOI:** 10.1186/s12935-020-01316-6

**Published:** 2020-06-10

**Authors:** Zhaoxian Lin, Xing Lin, Lihuan Zhu, Jianyuan Huang, Yangyun Huang

**Affiliations:** Department of Thoracic Surgery, Fujian Provincial Hospital, No. 134 East Street, Gulou District, Fuzhou, 350001 Fujian China

**Keywords:** Lung adenocarcinoma, EMT, TRIM2, Snail1, QRT-PCR

## Abstract

**Background:**

Lung adenocarcinoma has surpassed lung squamous cell carcinoma as the most common type of non-small cell lung cancer. In this study, we had tested the biological role of TRIM2 in lung adenocarcinoma.

**Methods:**

TRIM2 abundance in clinical tissues and six cell lines were examined with quantitative real-time PCR test (qRT-PCR) and western blot. TRIM2 overexpression treated H322 cells and TRIM2 knockdown treated A549 cells were used to study cell proliferation, migration, colony formation, invasion, and the expression of epithelial mesenchymal transformation (EMT) biomarkers. Moreover, ubiquitination related Snail1 degradation were studied with qRT-PCR and western blot. The relationships between TRIM2 and Snail1 were investigated with western blot, co-immunoprecipitation, migration, and invasion.

**Results:**

TRIM2 was highly expressed in lung adenocarcinoma tissues. TRIM2 overexpression and knockdown treatments could affect cell proliferation, colony formation, migration, invasion, and the expression of EMT associated biomarkers. Moreover, TRIM2 can regulate the ubiquitination related Snail1 degradation. In addition, TRIM2 can regulate Snail1 degradation in lung adenocarcinoma via ubiquitination pathway. TRIM2 could promote the proliferation, migration, and invasion of lung adenocarcinoma. Meanwhile, TRIM2 can deubiquitinate and stabilize Snail1 protein, which play important role in the function of lung adenocarcinoma.

**Conclusion:**

A high TRIM2 expression could be detected in lung adenocarcinoma tissues and cells. TRIM2 could aggravate cell proliferation, invasion, and migration in colorectal cancer by regulating Snail1 ubiquitylation degradation. Our results could provide detailed information for further studies in lung adenocarcinoma.

## Background

Lung cancer can be divided into non-small-cell cell lung cancer (NSCLC) and small cell lung cancer (SCLC), accounting for about 85% of the total number of lung cancers [1]. Meanwhile, lung cancer can also be categorized as three types according to its pathological characteristics: squamous cell carcinoma, adenocarcinoma, and large cell carcinoma [2]. Incidence of lung adenocarcinoma in China has gradually increased and has surpassed lung squamous cell carcinoma as the most common type of non-small cell lung cancer [3]. Lung adenocarcinoma treatment methods mainly include surgery, chemotherapy, radiotherapy, targeted therapy, biological therapy, etc., which can achieve better results in early patients. However, the overall effect on patients with advanced-stage is not ideal [4]. Chemotherapy is the main treatment method for patients with advanced lung adenocarcinoma. However, most patients are characterized by insensitivity to chemotherapy drugs, susceptibility to tumor metastasis, and poor prognosis [5]. Therefore, research on the molecular mechanism that affects patients with advanced lung adenocarcinoma has become one of the hot topics in lung adenocarcinoma research.

The TRIM (tripartite motif protein) family has more than 70 members. Previous studies have shown that TRIM family members play important roles in cell growth, differentiation, development, apoptosis, inflammation, and immunity [6]. The TRIM family of proteins contains a conserved “RBCC” motif, which includes the RING domain, the B-box motif, and the coiled-coil region [7]. Tri-domain protein 2 (tripartite motif-containing protein 2, TRIM2) belongs to the TRIM family of proteins, which is a kind of ring finger E3 ubiquitin. Previous studies have suggested that high expression of RIM2 is associated with neural activity in epilepsy. This molecule can participate in the regulation of neural cell mechanisms with myosin V [8]. Meanwhile, the lack of TRIM2 can reduce the ubiquitination of neurofilament lightsubstances (NF-L), which can cause neurodegenerative changes [9]. Moreover, TRIM2 can employ ubiquitination to degrade Bim (Bcl-2 interminding medial deafeat), which has been proven to be a regulatory mechanism of neuroprotection induced by rapid ischemic tolerance [10]. In cancer studies, recent studies have shown that TRIM2 is highly expressed in many primary diseases such as breast cancer, liver cancer, and viral hepatitis [11, 12]. The high expression of this molecule is related to tumor cell proliferation, apoptosis, metastasis, and tumor angiogenesis. Therefore, TRIM2 is considered as an oncogene [13]. However, the function of TRIM2 in lung adenocarcinoma has not been reported. Therefore, investigating the molecular mechanism of TRIM2 in the occurrence and development of lung adenocarcinoma provides an important clinical reference for the early diagnosis and prognosis evaluation of lung adenocarcinoma.

In this study, we had investigated the expression of TRIM2 in online databases, clinical samples, and cell lines. The functional roles of TRIM2 in cell lines had been further studied. Moreover, the detailed molecular mechanisms of TRIM2 and Snail1 were reported. The information provided in this study could provide valuable clues to further study in lung adenocarcinoma.

## Materials and methods

### Tissue samples and cells

Four lung adenocarcinoma samples and their paired normal tissues were collected in the Department of pathology, Fujian provincial hospital between Jan 2018 and Mar 2019. The ethics committee of Fujian provincial hospital had reviewed and approved all experimental protocols. All patients had read and signed the informed consent. The detached tissues were quickly frozen with fluid nitrogen and stored at – 80 °C. HNBE, H322, H1299, H460, H1703, and A549 cells were purchased from ATCC (Virginia, USA). Cells were cultured with RPMI 1640 with 10% (v/v) FBS (Invitrogen, Carlsbad, CA) in a humidified chamber at 5% CO_2_, at 37 °C. A549 cells were plated on six-well plates (5 × 10^5^ cells per well). DMEM with 10% FBS without penicillin and streptomycin overnight was used as a culture medium. OPTI-MEM serum-free medium (M5650, Sigma Aldrich) and Lipofectamine 2000 reagent (Thermo Fisher Scientific, USA) was used in transfection tests. The final concentration of 100 nM siRNA was introduced in this study. Meanwhile, pEZ-Lv201 Vector (Biovector, China) was employed to construct the TRIM2 over-expression system in H322 cells. pEZ-Lv201 Vector was used as the negative control in normal H322 cells. Lentiviral particles generated with a standardized protocol were used to produce the highly purified plasmids. Endo Fectin-Lenti™ and Titer Boost™ reagents (CWBio, China) were used to co-transfect H322 cells. The supernatant was collected after 48 h transfection and stored at − 80 °C.

### qRT-PCR analysis

Total RNA was extracted with M5 SuperPure Total RNA Extraction Reagent (SuperTRIgent) (mei5bio, China). The mRNA expression was examined with Q225 system (kubotechnology, China). The PCR reaction contained: 10 μL GoldStar Probe Mixture (Low ROX) (CWBio, China), 1 μL sense primer (10 nM), 1 μL anti-sense primer (10 nM), 2 μL cDNA template (10 ng), and 6 μL H_2_O. The program qRT-PCR was set as follows: 95 °C, 30 s, 40 cycles (95 °C, 5 s, and 60 °C, 10 s). 2^−ΔΔCt^ cycle method was used to calculate the relative expression level of mRNAs. GAPDH was employed as the internal control. Primer sequences were as follows: TRIM2: Forward Primer_CAGCATCCCAAGTCCCGTGGT, Reverse Primer_TCTCGCAGAAAGTGTGCAGACAG; GAPDH: Forward Primer_TGTGGGCATCAATGGATTTGG, Reverse Primer_ACACCATGTATTCCGGGTCAAT; Bmil: Forward Primer_TAAGTTCTGAGTGTGACCGAGA, Reverse Primer_GCTCTGTCTGTAGGGAGGTAGG; ALDH1: Forward Primer_CCGTGGCGTACTATGGATGC, Reverse Primer_GCAGCAGACGATCTCTTTCGAT; CD133: Forward Primer_AGTCGGAAACTGGCAGATAGC, Reverse Primer_GGTAGTGTTGTACTGGGCCAAT; Snail1: Forward Primer_TCGGAAGCCTAACTACAGCGA, Reverse Primer_AGATGAGCATTGGCAGCGAG; MMP9: Forward Primer_TGTACCGCTATGGTTACACTCG, Reverse Primer_GGCAGGGACAGTTGCTTCT; E-Cadherin: Forward Primer_CGAGAGCTACACGTTCACGG, Reverse Primer_GGGTGTCGAGGGAAAAATAGG; N-Cadherin: Forward Primer_TTTGATGGAGGTCTCCTAACACC, Reverse Primer_ACGTTTAACACGTTGGAAATGTG; Vimentin: Forward Primer_AGGCAAAGCAGGAGTCCACTGA, Reverse Primer_ATCTGGCGTTCCAGGGACTCAT; Cyclin D1: Forward Primer_GCTGCGAAGTGGAAACCATC, Reverse Primer_CCTCCTTCTGCACACATTTGAA; PCNA: Forward Primer_CCTGCTGGGATATTAGCTCCA, Reverse Primer_CAGCGGTAGGTGTCGAAGC.

### Western blot analysis

Cellular protein in different groups was extracted with 1% PMSF a RIPA Lysis and Extraction Buffer (Beyotime, China). After the total protein was reacted with SDS-PAGE test buffer, sodium dodecyl sulfate-polyacrylamide gel electrophoresis was used to perform further examination. In this step, the proteins were transmembrane onto a polyvinylidene difluoride layer (Novus, USA). After being blocked for 1 h at room temperature, the layer was brooded with anti-Rabbit TRIM2 (1:10,000) (20356-1-AP, Proteintech, USA), Snail1 (1:1000) (#3879, CST, USA), Bmil (1:1000) (#2742, CST, USA), ALDH1 (1:1000) (#54135, CST, USA), CD133 (1:1000) (#64326, CST, USA), GAPDH (1:1000) (#2118, CST, USA), Cyclin D1 (1:1000) (#2978 CST, USA), PCNA (1:1000) (#13110 CST, USA), E-Cadherin (1:1000) (#31958, CST, USA), N-Cadherin (1:1000) (#13116, CST, USA), Vimentin (1:1000) (#5741, CST, USA), and MMP9 (1:1000) (#13667, CST, USA) overnight. Proteins were hatched with the corresponding secondary antibodies for 1 h at room temperature after treated with ECL Chemiluminescence Detection Kit (PromoCell, German). The bands were observed with Chemiluminescence Imaging (clinx Ltd., China).

### Immunohistochemical staining analysis

The immunohistochemical SP method was used to stain cancer tissue sections. Tissue sections were baked in a 60 °C incubator for 1 h. The tissue sections were subjected to multiple treatments, including immersion in xylene to dewax, gradient alcohol hydration, microwave antigen repair, and 3% hydrogen peroxide treatment. After the goat serum was blocked, an anti-rabbit TRIM2 monoclonal antibody (1:600) (67342-1-Ig, Proteintech, USA) was added and incubated at 4 °C overnight. The section was observed under an optical microscope.

### Migration and invasion assay

EZCell™ Cell Migration/Chemotaxis Assay Kit (24-well) (K911-12, Biovision, USA) and EZCell™ Cell Invasion Assay (Basement Membrane) (96-well Kit) (K912-100, Biovision, USA) were used to performing cell migration assay and cell invasion, respectively. The detailed steps were strictly followed by the instruction provided by the manufacturer.

### Cck8

The differently treated cells were digested, centrifuged, and resuspended. The cells were diluted with complete medium. We counted using a cell glass counting plate, and then diluted the cells to 2000 cells/mL. 100 μL of a 2000 cells/mL cell suspension was added to each well in a 96-well plate. There are 5 replicate wells in each group. Five replicates were set and observed in five-time points. Subsequently, we incubated the cells in a 5% CO_2_, 37 °C incubator overnight. The next day, 10 μL of CCK-8 solution (Beyotime, China) was added to the medium of the first 5 pairs of wells. Then, the plate was incubated in a 37 °C incubator for 2 h. The absorbance at OD450 was measured after the crystals were thoroughly dissolved. Then, cell proliferation was calculated.

### Colony formation

Cells were inoculated in a 6-well plate with a density of 1000 cells/well, and cultured in 37 °C/5% CO_2_. The cell clone size was observed, and the medium was changed as appropriate according to the medium condition. When macroscopic clones appeared, the culture was terminated. The medium in the well was discarded. The well was washed twice with PBS, and was air-dried. Cells were then fixed with 4% paraformaldehyde for 30 min. After drying, it was stained with 1% crystal violet dye solution for 30 min. subsequently, cell colony formation was observed under an optical microscope.

### Scratch test

We resuspended and counted different TRIM2 treated cells. The scratch test insert after alcohol disinfection was carefully placed in a 12-well plate (3 replicates per group). We used complete medium to dilute the cells to 500 cells/μL. 70 μL of the cell suspension was added to each well. Place at 37 °C (incubate in a 5% CO_2_ incubator for 24 h). After 24 h, we gently washed the cells twice with PBS. Then, 1 mL of 1% FBS corresponding medium was added. Cell status was observed under the microscope at 0 h and 24 h.

### Cell pelleting

We used DMEM/F12 medium containing 20 ng/mL of EGF, 20 ng/mL bFGF, and B27 for cell pelleting experiments. We resuspended and counted the different TRIM2 treated cells. 1 mL of medium and 200 cells were added to a low-adhesion 24-well plate. 100 μL of the above-mentioned medium was added every 1 day. After 2 weeks, the picture was taken under a microscope. The diameter of the cell pellet was measured and counted.

### Immunofluorescence analysis

Cancer cells in the logarithmic growth phase were inoculated into 24-well plates with cell slides and cultured for 48 h. We discarded the medium, removed the cell slides, and washed 3 times with PBS. Sections were fixed with 4% paraformaldehyde at 4 °C for 30 min. We have washed 3 times at room temperature with PBS for 5 min each. Furthermore, 0.1% Triton was used to treat sections for 10 min and PBS was used to wash sections for 5 min. The goat serum incubation section was blocked for 1 h at room temperature. The goat serum incubation section was blocked for 1 h at room temperature. Subsequently, we added a diluted secondary antibody, reacted for 1 h at room temperature in a wet box, and washed 3 times with PBS for 10 min each. We use an inverted fluorescence microscope to observe the results. The staining scores were validated by an experienced pathologist. Five filed in each groups were evaluated by the experienced pathologist, and staining scores were calculated as described previously [14]. The staining scores were calculated based on the following rules: very strong (200–300), strong (100–200), weak (40–100), absent (0–40).

### Co-IP detection

In this study, we use cancer cells in logarithmic growth phase. Total protein was extracted using the RIPA Lysis and Extraction Buffer (89900, ThermoFisher Scientific, USA). In short, we washed the beads with 100 μL of ice-cold buffer. We added 100 μL of antibody binding buffer to spin the antibody and magnetic beads for 30 min. We washed the beads 3 times with 200 μL of buffer for 5 min each. We used cell lysate and antibody-conjugated magnetic beads to incubate for 1 h at room temperature and washed the beads 3 times with 200 μL buffer for 5 min each. 20 μL of elution buffer was used to wash the beads once and the supernatant was taken.

### Statistical methods

In this study, SPSS16.0 statistical software was used. The data was expressed as χ ± s. Two groups were compared using t test. One-way analysis of variance was used for comparison between groups. P values < 0.05 were considered to be significant differences between the two data.

## Results

### TRIM2 gene is highly expressed in lung adenocarcinoma tissue

**Fig. 1:**
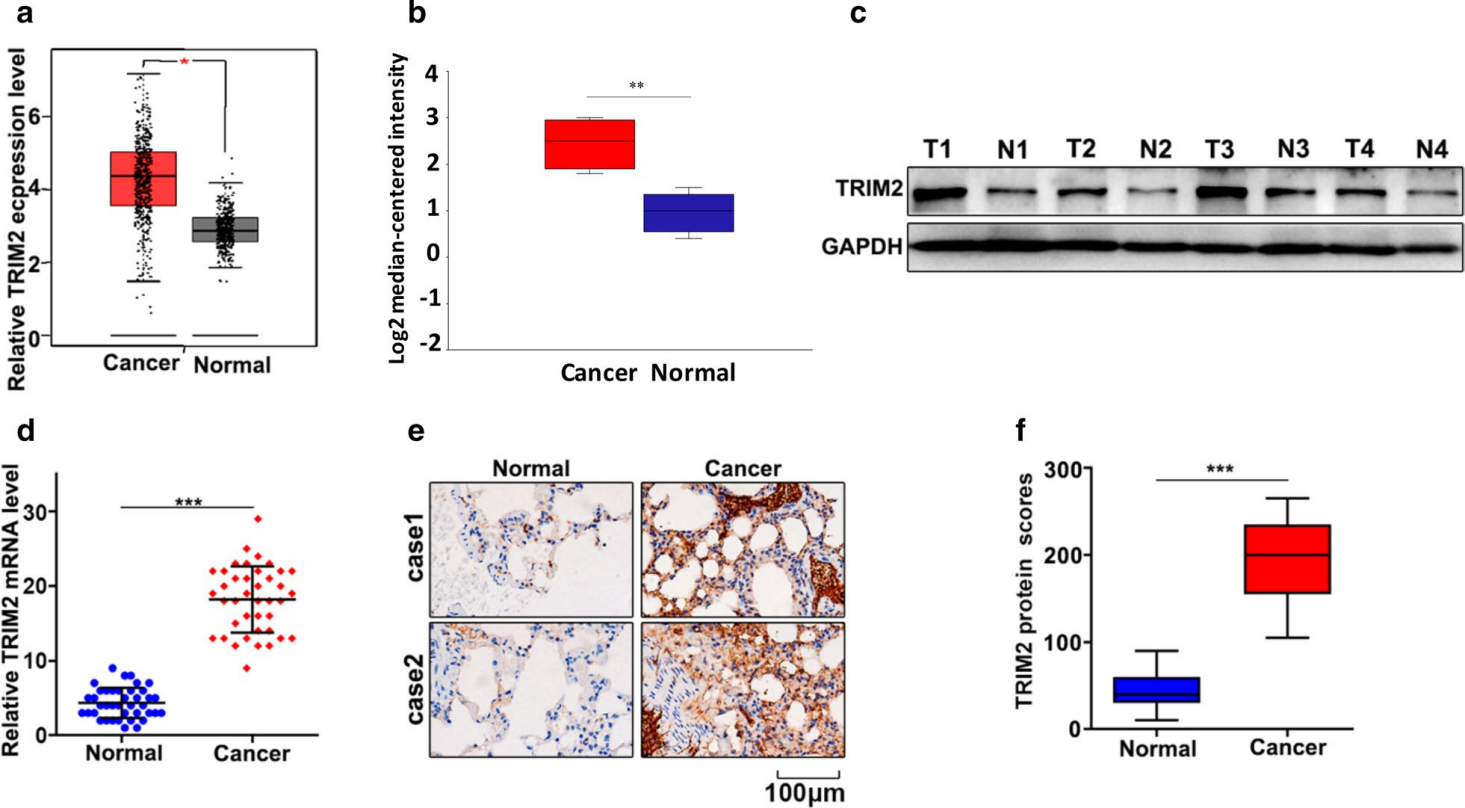
TRIM2 gene is highly expressed in lung adenocarcinoma tissues. **a** mRNA abundance analysis of TRIM2 gene in GEPIA database. **b** mRNA abundance analysis of TRIM2 gene in oncomine database. **c** Western blot analysis of normal tissues and ung adenocarcinoma tissues. N: normal tissues; T: tumor tissues. GAPDH was employed as an internal reference. **d** qRT-PCR analysis the TRIM2 mRNA abundance in four lung adenocarcinoma tissues and paired normal tissues. **e**, **f** Immunohistochemical analysis of normal tissues and lung adenocarcinoma tissues. ***P < 0.001

Firstly, we had detected the expression of TRIM2 in lung adenocarcinoma tissue and the paired normal tissues with the online dataset, western blot, qRT-PCR, and immunohistochemical staining analysis. For online dataset analysis, we evaluated TRIM2 expression in GEPIA database (http://gepia.cancer-pku.cn/index.html) and Oncomine database (https://www.oncomine.org/). TRIM2 expression in lung adenocarcinoma tissues was higher than those in the paired normal tissues (P < 0.05) (Fig. 1a, b). Meanwhile, western blot and qRT-PCR analysis indicated that TRIM2 expression in lung adenocarcinoma tissues was significantly higher than that in the paired normal tissues (Fig. 1c, d). Moreover, immunohistochemical staining analysis indicated that TRIM2 expression in lung adenocarcinoma tissue was significantly higher than that in the paired normal tissues (Fig. 1e, f). In summary, TRIM2 expression in lung adenocarcinoma tissue was higher than those in the paired normal tissues.

### TRIM2 promotes proliferation of lung adenocarcinoma cells in vitro

**Fig. 2:**
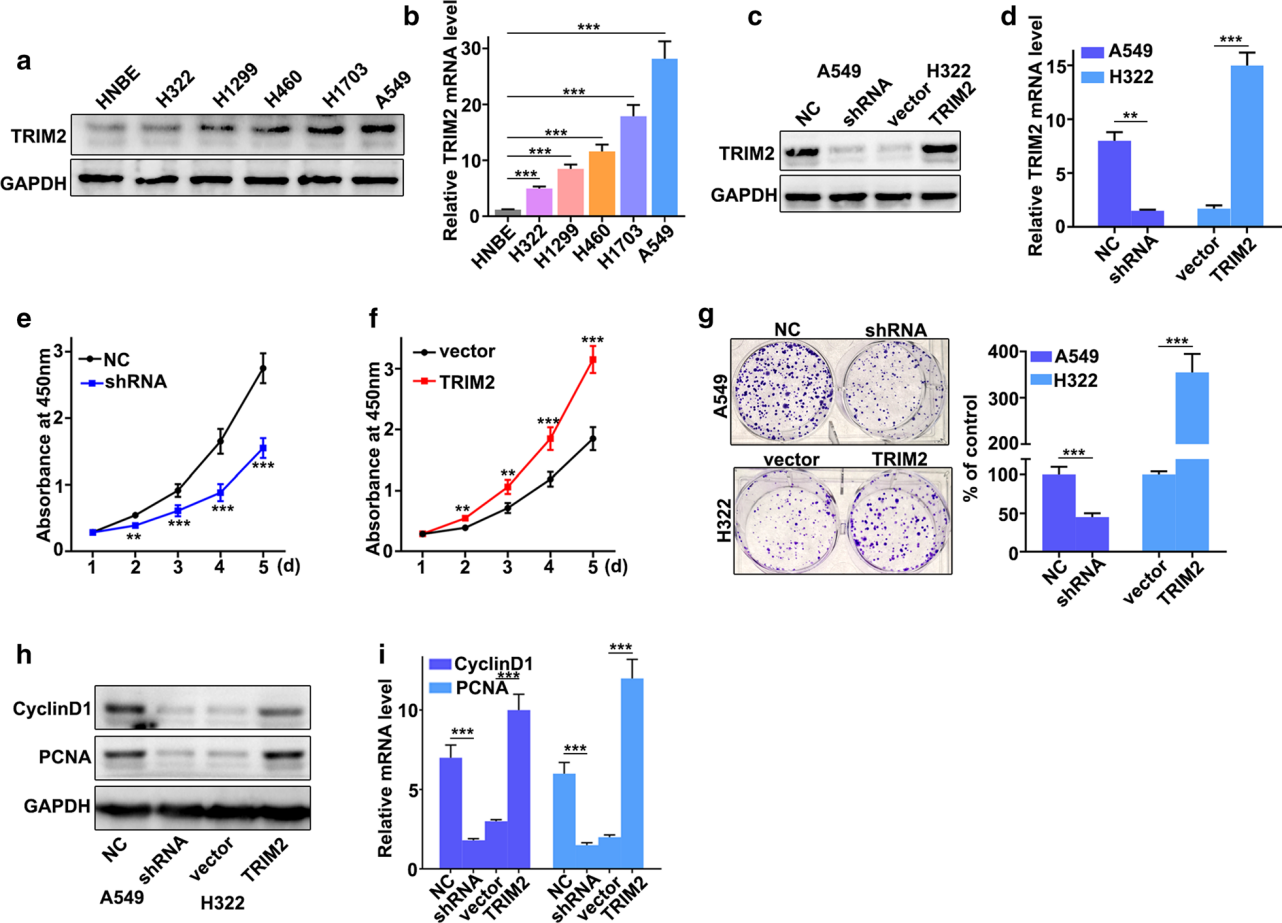
TRIM2 promotes proliferation of lung adenocarcinoma cells in vitro. **a** Western blot analysis of TRIM2 expression in HNBE, H322, H1299, H460, H1703, and A549 cells. **b** qRT-PCR analysis of TRIM2 expression in HNBE, H322, H1299, H460, H1703, and A549 cells. **c** TRIM2 protein expression of the TRIM2 knockdown treated A549 cells and TRIM2 overexpression treated H322 cells. **d** TRIM2 mRNA expression of the TRIM2 knockdown treated A549 cells and TRIM2 overexpression treated H322 cells. **e** Cell proliferation analysis of TRIM2 knockdown treated A549 cells. **f** Cell proliferation analysis of TRIM2 overexpression treated H322 cells. **g** Colony formation analysis of the TRIM2 knockdown treated A549 cells and TRIM2 overexpression treated H322 cells. **h**, **i** Western blot analysis of cell proliferation-related biomarkers expression in TRIM2 knockdown treated A549 cells and TRIM2 overexpression treated H322 cells.**P < 0.01, ***P < 0.001

In order to further probe the biological function of TRIM2 in lung adenocarcinoma cells, we have studied TRIM2 expressions in six selected cell lines, including HNBE, H322, H1299, H460, H1703, and A549. Western blot analysis and qRT-PCR analysis of TRIM2 expression in six cell lines indicated that TRIM2 protein and mRNA expressions were significantly different between each other (Fig. 2a, b). It was notable that TRIM2 protein and mRNA expression in H322 cells were lower than those in other cell lines (P < 0.01). Meanwhile, TRIM2 protein and mRNA expression in A549 cells were higher than those in other cell lines (P < 0.001). Therefore, H322 and A549 cells were selected to carry out further study. For H322 and A549 cells, we performed overexpression and knockdown of TRIM2 treatments, respectively. We have employed western blot and qRT-PCR analysis to examine the TRIM2 protein and mRNA expression in H322 and A549 cells with different treatments. Figure 2c, d showed that overexpression and knockdown of TRIM2 treatments in H322 and A549 cells were successful. For cell proliferation analysis, TRIM2 knockdown in A549 cells could significantly reduce cell proliferation compared with those in normal A549 cells on day 2, 3, 4, 5 (Fig. 2e) (P < 0.01). However, TRIM2 overexpression in H322 cells could significantly promote cell proliferation compared with those in vector treated H322 cells on day 2, 3, 4, 5 (Fig. 2f) (P < 0.01). Moreover, TRIM2 overexpression in H322 cells could significantly increase cell colony numbers compared with those in vector treated H322 cells (Fig. 2g) (P < 0.01). However, TRIM2 knockdown in A549 cells could significantly decrease cell colony numbers compared with those in normal A549 cells (Fig. 2g) (P < 0.01). Meanwhile, cell proliferation-related biomarkers such as CyclinD1 and PCNA in TRIM2 knockdown of A549 cells and TRIM2 overexpression of H322 cells were studied with western blot and qRT-PCR. The results suggested that the protein and mRNA expressions of CyclinD1 and PCNA in TRIM2 knockdown of A549 cells were lower than those in normal A549 cells (P < 0.05) (Fig. 2h, i). However, the protein and mRNA expressions of CyclinD1 and PCNA in TRIM2 overexpression of H322 cells were higher than those in normal H322 cells (P < 0.05) (Fig. 2h, i). In summary, TRIM2 over-expression in H322 cells could promote cell proliferation of lung adenocarcinoma cells in vitro. Meanwhile, TRIM2 knockdown in A549 cells can restrain cell proliferation of lung adenocarcinoma cells in vitro.

### Regulation of TRIM2 on lung adenocarcinoma cell migration and invasion

**Fig. 3:**
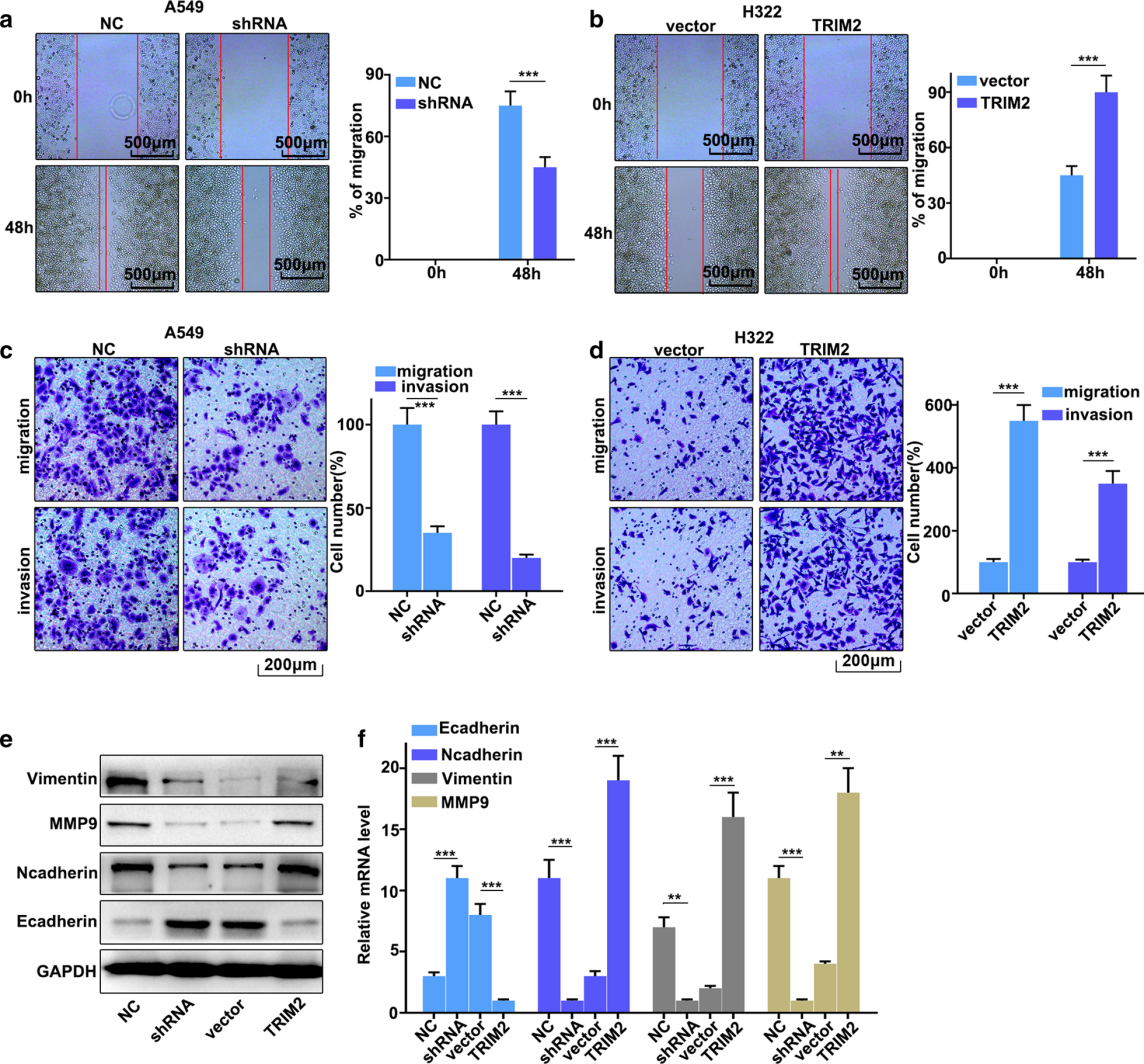
Regulation of TRIM2 on lung adenocarcinoma cell migration and invasion. **a** Cell scratch test of TRIM2 knockdown treated A549 cells. **b** Cell scratch test of TRIM2 overexpression treated H322 cells. **c**, **d** Cell migration and invasion analysis of TRIM2 knockdown treated A549 cells and TRIM2 overexpression treated H322 cells, respectively. **e** Western blot analysis of EMT-related biomarkers expression in TRIM2 knockdown treated A549 cells and TRIM2 overexpression treated H322 cells. **f** qRT-PCR analysis of EMT-related biomarkers expression in TRIM2 knockdown treated A549 cells and TRIM2 overexpression treated H322 cells. **P < 0.01, ***P < 0.001

In this work, we have investigated the regulation of TRIM2 on lung adenocarcinoma cell migration and invasion in TRIM2 over-expression treated H322 cells and TRIM2 knockdown treated A549 cells. Figure 3a showed that TRIM2 knockdown treatment could significantly inhibit cell scratch ability compared with those in normal A549 cells (P < 0.01). Meanwhile, TRIM2 overexpression in H322 cells could significantly promote cell scratch ability compared with those in normal H322 cells (P < 0.01) (Fig. 3b). Moreover, we have further examined the cell migration and invasion in TRIM2 over-expression treated H322 cells and TRIM2 knockdown treated A549 cells. TRIM2 knockdown in A549 cells could significantly inhibit cell migration and invasion compared to that in the normal A549 cells (P < 0.01) (Fig. 3c). However, TRIM2 overexpression in H322 cells could promote cell migration and invasion compared to that in the normal H322 cells (P < 0.01) (Fig. 3d). In addition, we have further analysis of the protein expressions of E-cadherin, N-cadherin, Vimentin, and MMP9. TRIM2 knockdown treatment in A549 cells could effectively inhibit protein expressions of N-cadherin, Vimentin, and MMP9 (P < 0.01) (Fig. 3e, f). However, TRIM2 knockdown treatment in A549 cells could effectively promote protein expressions of E-cadherin (P < 0.01). Moreover, TRIM2 overexpression in H322 cells could effectively increase protein expressions of N-cadherin, Vimentin, and MMP9 (P < 0.01) (Fig. 3e, f). However, TRIM2 overexpression in H322 cells could effectively inhibit E-cadherin protein expressions (P < 0.01). Therefore, TRIM2 overexpression in H322 cells could promote and inhibit the protein expressions of N-cadherin, MMP9, and Vimentin and protein expressions of N-cadherin, respectively. Meanwhile, TRIM2 knockdown in A549 cells could inhibit and promote the protein expressions of N-cadherin, MMP9, and Vimentin and protein expressions of N-cadherin, respectively.

### Effect of TRIM2 on chemotherapy sensitivity of lung adenocarcinoma cells

**Fig. 4:**
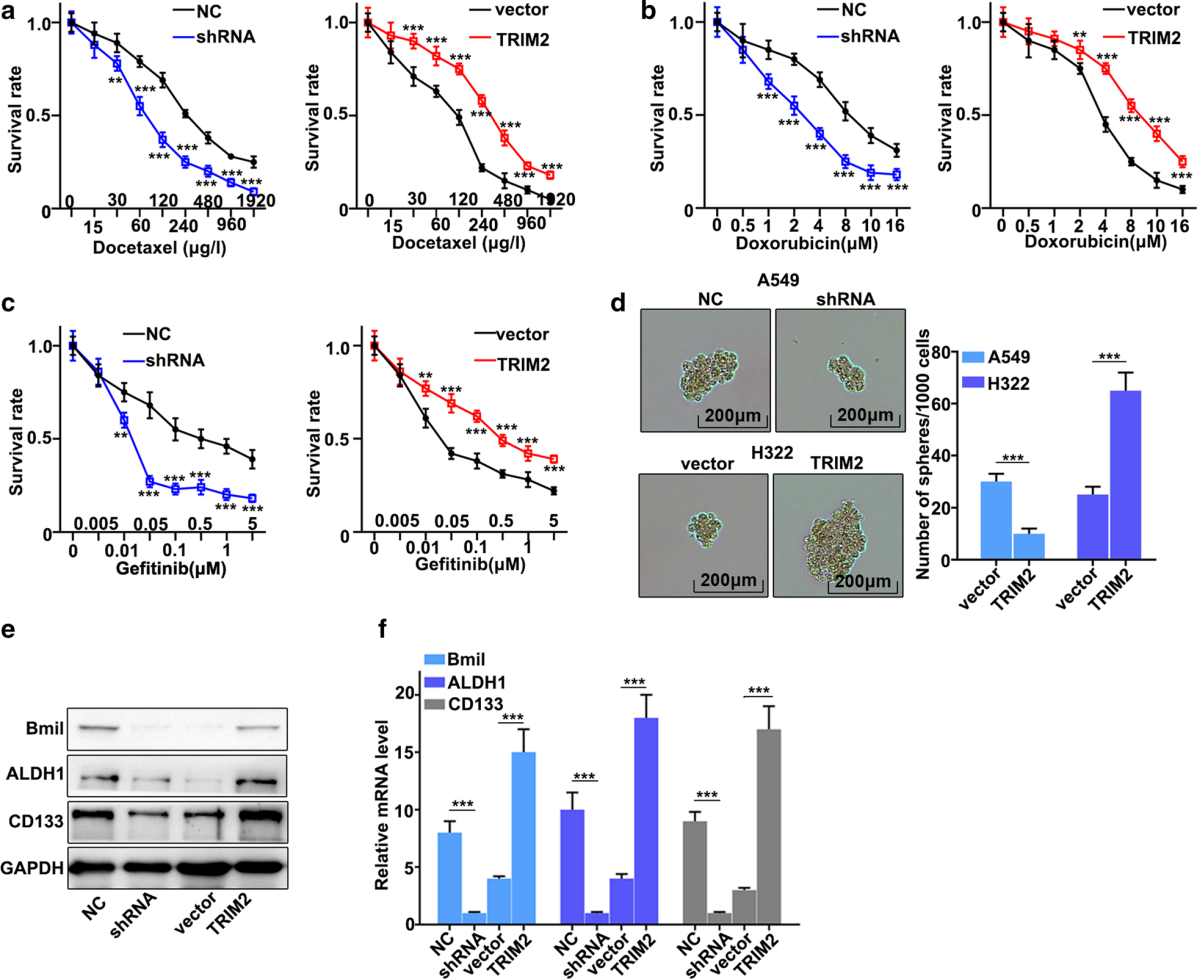
Effect of TRIM2 on chemotherapy sensitivity of lung adenocarcinoma cells. **a**–**c** cell survival analysis of three chemotherapy drugs in TRIM2 knockdown treated A549 cells and TRIM2 overexpression treated H322 cells. **d** Stem cell pellet culture analysis of TRIM2 knockdown treated A549 cells and TRIM2 overexpression treated H322 cells. **e** Western blot analysis of stem cell-related biomarkers. **f** qRT-PCR analysis of stem cell-related biomarkers. **P < 0.01, ***P < 0.001

In this study, we have investigated the effect of TRIM2 expression changes on chemotherapy sensitivity of lung adenocarcinoma cells. We have employed three common chemotherapeutic molecular drugs, including docetaxel, doxorubicin, and gefitinib to probe the survival rate of TRIM2 over-expression treated H322 cells and TRIM2 knockdown treated A549 cells. Figure 4a–c showed the survival rate of TRIM2 over-expression treated H322 cells and TRIM2 knockdown treated A549 cells with different drug treatments. The results suggested that TRIM2 knockdown in A549 cells could significantly decrease cell survival in three different drug treatments compared with that in normal A549 cells (P < 0.05). However, TRIM2 over-expression treated H322 cells could effectively promote cell survival in three different drug treatments compared with that in normal H322 cells (P < 0.05). These results revealed that TRIM2 expression was closely related to the changes in cell survival rate caused by drug treatments. Moreover, we have performed stem cell pellet culture to study the effect of TRIM2 expression changes. Figure 4d showed that TRIM2 over-expression in H322 cells could effectively inhibit cell pellet formation (P < 0.001). Whereas, TRIM2 knockdown in A549 cells could effectively promote cell pellet formation (P < 0.001). In addition, we have evaluated the protein and mRNA expression of stem cell-related biomarkers, including Bmil, ALDH1, and CD133. Figure 4e, f indicated that TRIM2 knockdown in A549 cells could significantly decrease protein and mRNA expression of stem cell-related biomarkers (P < 0.001). However, TRIM2 over-expression in H322 cells could effectively promote protein and mRNA expression of stem cell-related biomarkers (P < 0.001). The above results suggested that TRIM2 expression was closely related to stem cell ability.

### TRIM2 regulates Snail1 expression through ubiquitination

**Fig. 5:**
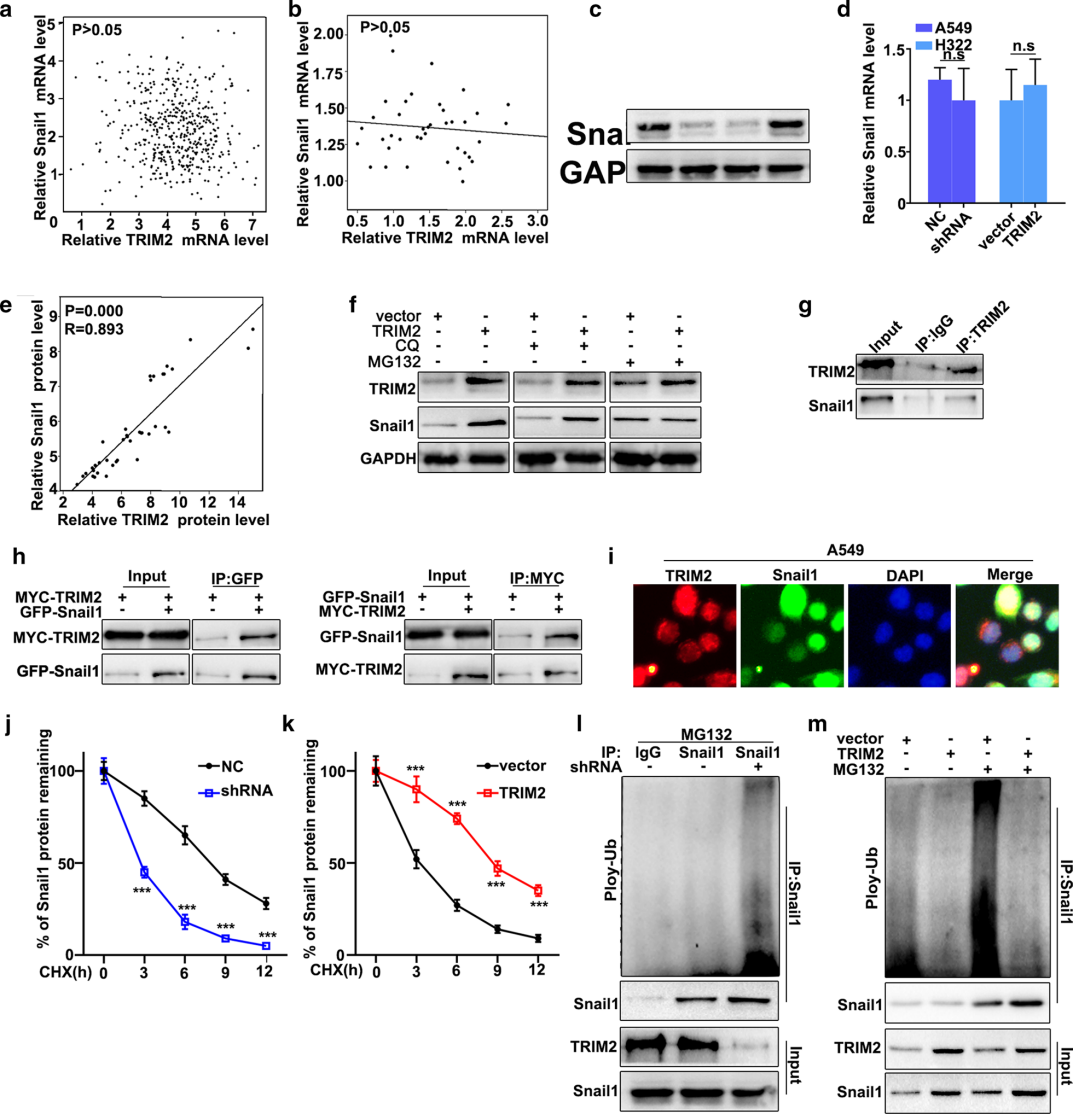
TRIM2 regulates Snail1 expression through ubiquitination. **a** Correlation analysis of TRIM2 mRNA and Snail1 mRNA in GEPIA database. **b** Correlation analysis of TRIM2 mRNA and Snail1 mRNA in clinical samples. **c** Western blot analysis of Snail1 protein expression in TRIM2 knockdown treated A549 cells and TRIM2 overexpression treated H322 cells. **d** qRT-PCR analysis of Snail1 protein expression in TRIM2 knockdown treated A549 cells and TRIM2 overexpression treated H322 cells. **e** Correlation analysis of TRIM2 protein expression and Snail1 protein expression in clinical samples. **f** Western blot analysis of TRIM2 and Snail1 expression in TRIM2 over-expression treated H322 cells with CQ and MG132 treatment on different time points. **g** CO-IP analysis of TRIM2 and Snail1 interaction in knockdown treated A549 cells. **h** Forward and reverse CO-IP analysis of TRIM2 and Snail1 in H322 cells and A549 cells. **i** Immunocolocalization analysis of TRIM2 and Snail1 in H322 cells and A549 cells. **j**, **k** Rrmaining Snail1 protein in H322 cells and A549 cells with cyclohexane (CHX) treatment on different time points. **l**, **m** Ubiquitination test of TRIM2 and Snail1 in A549 cells and H322 cells, respectively. ***P < 0.001

In this study, we have further studied the potential relationships between the expressions of TRIM2 regulates Snail1. We have further analyzed the potential relationships of the mRNA expressions of TRIM2 and Snail1 in GEPIA online database and clinical samples (Fig. 5a, b). The results suggested that no correlation could be calculated between the mRNA expressions of TRIM2 and Snail1 in GEPIA online database and clinical samples. Moreover, western blot and qRT-PCR were employed to investigate the protein and mRNA expressions of Snail1 in TRIM2 over-expression treated H322 cells and TRIM2 knockdown treated A549 cells. Figure 5c showed that Snail1 protein expression in TRIM2 knockdown treated A549 cells was lower than that in normal A549 cells. However, Snail1 protein expression in TRIM2 overexpressed H322 cells was more abundant compared with that in normal H322 (Fig. 5c). It was notable that no difference of Snail1 mRNA abundance could be detected in TRIM2 over-expression treated H322 cells and TRIM2 knockdown treated A549 cells (Fig. 5d). The above results suggested that TRIM2 could affect the Snail1 protein expression but not Snail1 mRNA abundance. The relationship analysis between TRIM2 protein and Snail1 protein suggested that they had significantly correlation (P = 0.000) (Fig. 5e). Moreover, we have investigated the potential relationships between TRIM2 ubiquitination and Snail1 in TRIM2 overexpression H322 cells. MG132 is the inhibitor of the proteasome degradation pathway in the cell. Meanwhile, Chloroquine (CQ) is an inhibitor of the autophagolysosomal degradation pathway. Therefore, we have employed MG132 (25Um) and CQ (25Um) to study the protein expressions of TRIM2 and Snail1 in TRIM2 overexpression H322 cells (Fig. 5f). The results suggested that MG132 can effectively reduce Snail1 protein expression. However, CQ treatment had no effect on Snail1 protein expression. Therefore, we speculate that TRIM2 could regulate Snail1 expression through the ubiquitination but not the cellular autophagy pathway. Moreover, we have examined the potential interaction between TRIM2 protein and Snail1 protein in cellular. In this study, Co-immunoprecipitation (Co-IP) was employed to study the potential interaction between TRIM2 protein and Snail1 protein. Figure 5g showed that TRIM2 protein could interact with Snail1 protein. Moreover, we have carried out the forward and reverse protein Co-IP of TRIM2 protein and Snail1 protein in TRIM2 overexpression H322 cells. Figure 5h had further identified the interaction between TRIM2 protein and Snail1 protein. Furthermore, immunofluorescence colocalization analysis of TRIM2 protein and Snail1 protein revealed that the spatial distribution of TRIM2 and Snail1 is overlapping. The above result further illustrates the mutual combination between TRIM2 and Snail1 in TRIM2 overexpression H322 cells (Fig. 5i). Furthermore, we have further employed cycloheximide (CHX), which could to inhibit protein synthesis to study the relationships between protein expressions of TRIM2 and Snail1 in TRIM2 over-expression treated H322 cells and TRIM2 knockdown treated A549 cells. Figure 5j, k suggested that TRIM2 overexpression in H322 cells could significantly promote the remaining Snail1 protein in cells compared to that normal H322 cells (P < 0.01). Meanwhile, TRIM2 knockdown A549 cells could significantly decrease the remaining Snail1 protein in cells compared to that normal A549 cells (P < 0.01). In addition, we have carried out ubiquitination tests in TRIM2 over-expression treated H322 cells and TRIM2 knockdown treated A549 cells. Figure 5l showed that TRIM2 knockdown treated A549 cells inhibits Snail1 ubiquitination degradation. Meanwhile, TRIM2 over-expression treated H322 cells could promote Snail1 ubiquitination degradation (Fig. 5m). In summary, TRIM2 can regulate Snail1 gene expression through ubiquitination.

### Rescue experiment

**Fig. 6:**
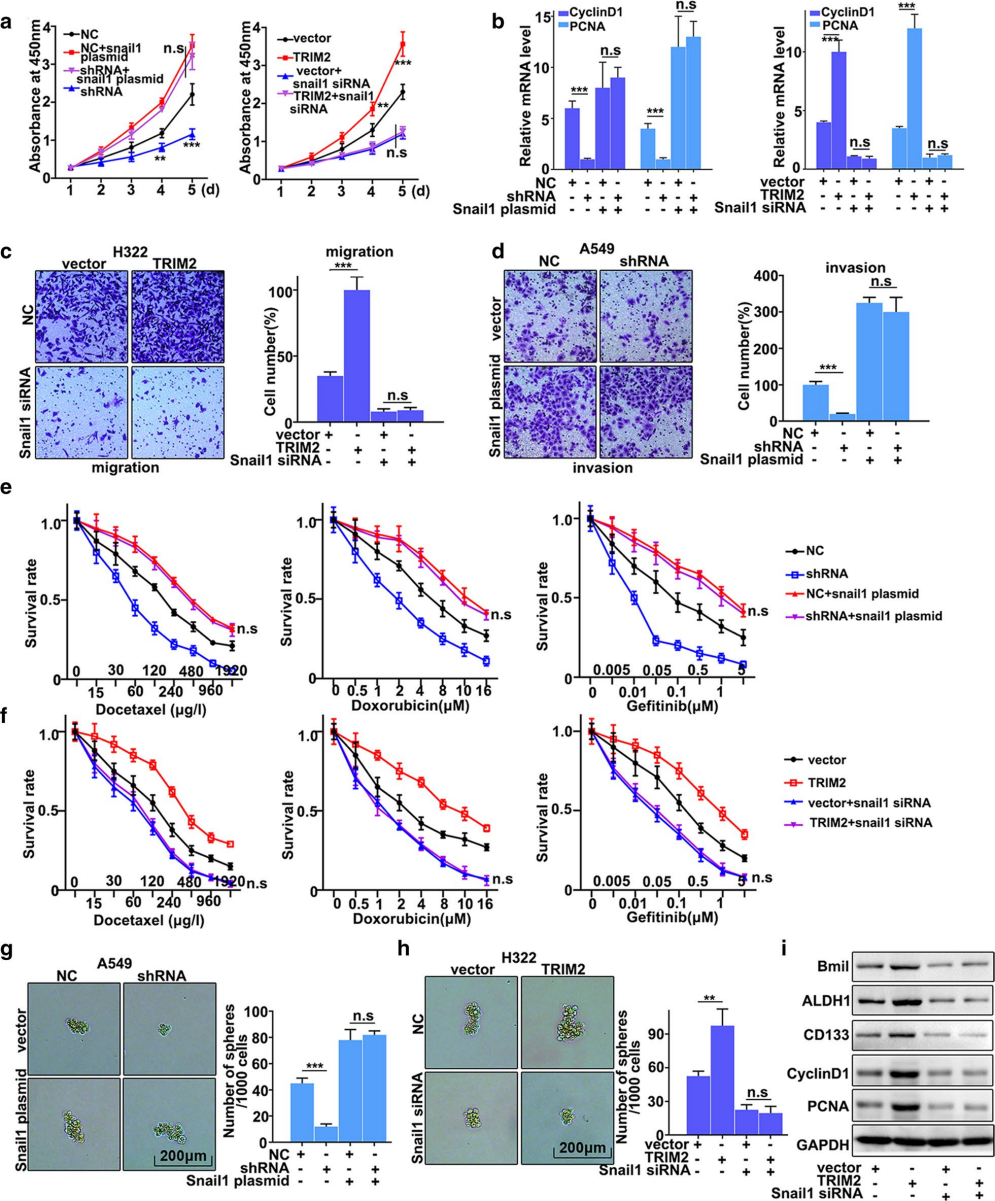
Rescue experiment. **a** CCK8 analysis of TRIM2 knockdown treated A549 cells and TRIM2 overexpression treated H322 cells that had been treated with Snail1 knockdown and overexpression treatments. **b** qRT-PCR analysis of Cyclin D and PCNA expression in TRIM2 knockdown treated A549 cells and TRIM2 overexpression treated H322 cells that had been treated with Snail1 knockdown and overexpression treatments. **c**, **d** Migration and invasion analysis of TRIM2 knockdown treated A549 cells and TRIM2 overexpression treated H322 cells that had been treated with Snail1 knockdown and overexpression treatments. **e**, **f** cell survival analysis of three chemotherapy drugs in TRIM2 knockdown treated A549 cells and TRIM2 overexpression treated H322 cells that had been treated with Snail1 knockdown and overexpression treatments. **g**, **h** Stem cell pellet analysis of the TRIM2 knockdown treated A549 cells and TRIM2 overexpression treated H322 cells that had been treated with Snail1 knockdown and overexpression treatments. **i** Wesstern blot analysis of stem cell related biomarkers in TRIM2 knockdown treated A549 cells and TRIM2 overexpression treated H322 cells that had been treated with Snail1 knockdown and overexpression treatments. ***P < 0.001

In this study, we have further performed the rescue experiment to demonstrate the relationships between Snail1 and TRIM2. We have further introduced Snail1 knockdown and over-expression treatment in TRIM2 overexpressed H322 cells and TRIM2 knockdown A549 cells. Figure 6a showed that Snail1 knockdown and over-expression treatment could effectively promote and inhibit cell proliferation changes caused by TRIM2 overexpression and knockdown treatments, respectively (Fig. 6a). Moreover, we have further examined the mRNA expressions of Cyclin D1 and PCNA, which were biomarkers for cell proliferation. Figure 6b suggested that Snail1 knockdown and over-expression treatment could effectively promote and inhibit the expressions of Cyclin D1 and PCNA changes caused by TRIM2 overexpression and knockdown treatments, respectively. Furthermore, we have studied the cell migration and invasion of the Snail1 knockdown and over-expression treatments, which had been treated with TRIM2 overexpression and TRIM2 knockdown, respectively. Figure 6c, d showed that Snail1 over-expression could effectively reverse the changes in migration and invasion caused by TRIM2 knockdown (P < 0.01). Meanwhile, Snail1 knockdown could effectively reverse the changes in migration and invasion caused by TRIM2 overexpression (P < 0.01) (Fig. 6c, d). Moreover, we have studied the effect of TRIM2 and Snail1 on chemotherapy sensitivity of lung adenocarcinoma cells. Figure 6e, f suggested that Snail1 over-expression and knockdown treatments could effectively reverse the changes in cell survival rate caused by TRIM2 knockdown and overexpression (P < 0.01). Figure 6g, h showed that Snail1 over-expression and knockdown treatments could effectively reverse the changes in cell pellet formation caused by TRIM2 knockdown and overexpression treatments (P < 0.01). A similar situation could be detected in mRNA abundance analysis (Additional file 1: Figure S1). In addition, we have detected the protein expressions of E-cadherin, N-cadherin, Vimentin, and MMP9, which were the biomarkers of EMT, in different treated groups. Figure 6i showed that Snail1 knockdown treatment could effectively reverse the protein expression changes of EMT related biomarkers caused by TRIM2 overexpression (P < 0.01). Meanwhile, Snail1 overexpression could effectively reverse the protein expression changes of EMT related biomarkers caused by TRIM2 knockdown treatment (P < 0.01). A similar situation could be detected in mRNA abundance analysis (Additional file 1: Figure S1). In summary, knockdown and overexpression of Snail1 could effectively rescue the changes in cell proliferation, migration, invasion, and EMT in TRIM2 overexpression and knockdown treatments.

## Discussion

Lung cancer is one of the most common malignancies in the world. The morbidity and mortality of the disease ranks first among various tumors [15]. Although cancer treatments, including lung adenocarcinoma, are constantly being updated and great progress has been made, the prognosis is still not ideal [16]. Meanwhile, most known anti-tumor drugs have a large toxic side effect on normal cells [17]. Therefore, The research on the mechanism of the development of lung adenocarcinoma is particularly important. It can not only help to understand the pathogenesis of lung adenocarcinoma, but also assist in the development of anti-tumor drugs.

Ubiquitination plays an important role in the regulation of almost all life activities. Among them, ubiquitination also plays an important role in the occurrence and development of various tumors [18]. For example, VHL (vonhippel-lindau) is considered a tumor suppressor gene in many tumors. This molecular is an E3 ubiquitin ligase that can bind to hypoxia-inducible factors-1α and -2-α (HIF-1α and HIF-2α) to cause degradation of HIF-α [19]. Therefore, the molecule can inhibit the proliferation of tumor cells [20]. The process of ubiquitination needs the involvement of enzymes suc as E1, E2 and E3. TRIM proteins are E3 ubiquitin ligases, and have been proved to be colesly related with the process of ubiquitination [12]. TRIM62 could promote the ubiquitination of SMAD3, which is the major target of EMT. Meanwhile, TRIM62 was believed to be a prognostic and predictive marker for breast cancers [21]. Snail1 is an important transcription factor of EMT, and the expression level of it is linked with the invasion, migration, and apoptosis of tumor cells. Snail1 is a labile protein, and the ubiquitination of it was believed to be regulated by several molecules including FBXO11, β-TRCP1, GSK3β, and FBXL14 [22, 23]. Whether TRIM2 could affect the ubiquitination and degradation of snail1 remain unclear.

Previous studies predict that TRIM2 may inhibit the proliferation and metastasis of renal cancer by degrading HIF-α [24]. Tumor stem cells are actually only a small part of the tumor. As long as chemoradiation does not kill all cancer stem cells, there is a risk of resistance or recurrence [25]. In this study, our results suggested that the change of TRIM2 expression was closely related to the invasion, migration, proliferation, tumor stemness and tumor stem cell spheroidizing ability of lung adenocarcinoma cells. Therefore, TRIM2 may provide a potential target for the treatment of lung adenocarcinoma. Previous study had shown that TRIM2 is differentially expressed in ovarian, cervical, follicular, and osteosarcoma tumors [13, 26, 27]. In studies of ovarian and follicular cancer, the expression of TRIM2 was detected to be down-regulated, which is inconsistent with the results of this study. However, in cervical cancer and osteosarcoma, upregulation of TRIM2 expression is consistent with this study. This results demonstrated the flexible role of TRIM2 in different cancer types. It was notable that upregulation of TRIM2 expression in rectal cancer can promote metastasis of rectal cancer cells through epithelial-mesenchymal transition [28]. This result was consistent to our results. Although the underlying mechanism of TRIM2 may be complex and diverse, it is still an important gene involved in tumorigenesis and development. This study showed that the expression of TRIM2 in lung adenocarcinoma tissues was higher than in adjacent tissues. Overexpression of TRIM2 or knockout of TRIM2 can affect the proliferation, migration and stemness of lung adenocarcinoma cells. Therefore, it is suggested that TRIM2 dysfunction may be one of the factors affecting the progression of lung adenocarcinoma.

Tumor metastasis requires the ability of tumor cells to penetrate the basement membrane. At present, it is believed that the induction of basal membrane invasion-related phenotypes is related to the epithelial transformation of tumor cells. This process allows epithelial-derived tumor cells to obtain an invasive and mesenchymal-like phenotype that can invade around the primary site. As a result, cancer cells can enter blood vessels and lymphatic vessels and cause distant metastases [29]. Therefore, EMT is the most important biological process in tumor metastasis, which is related to the tumor’s invasive characteristics and poor prognosis [30]. Among many regulators of EMT, snail1 is considered to be a key factor that not only participates in the development of mesoderm and neural tube during the embryonic period, but also plays an important role in tumor metastasis [31]. Snail1 is the most important E-cadherin transcriptional repressor. It can be combined with the E-box sequence of the E-cadherin promoter to suppress its transcription. In addition, Snail1 also down-regulates the expression of claudins and occludins protein through the same mechanism. Therefore, Snail1 can disrupt cell-to-cell adhesion [32]. Decreased or absent expression of these adhesion molecules is considered a marker of EMT and the first step in tumor invasion and metastasis. Previous studies have confirmed that the high expression of Snail1 and the down-regulation of E-cadherin expression are related to tumor growth, recurrence, metastatic tendency and poor prognosis. Knockout of Snail can significantly increase the expression of E-cadherin and down-regulate the expression of α-SMA and MMPs. Snail1 expression changes can regulate cell morphology, tumor invasion ability, and the growth of primary tumors [33]. In this study, it was notable that Snail1 (EMT biomarker) expression was significantly affected in TRIM2 overexpression and knockdown treatments. Snail1 can directly interact with TRIM2 in cellular. Moreover, TRIM2 can reduce Snail protein expression without affecting its transcription. Moreover, our results suggested that TRIM2 does affect the protein degradation pathway of Snail through ubiquitination modification. Meanwhile, TRIM2 overexpression and knockdown treatments with knockdown and overexpression of Snail1 could affect cell migration, invasion, and EMT-related biomarkers such as E-cadherin, N-cadherin, Vitmentin, and MMP9. Therefore, our results revealed that TRIM2 and Snail can regulate EMT of lung adenocarcinoma.

## Conclusion

In summary, we demonstrated that high TRIM2 expression could be detected in lung adenocarcinoma tissues and cells. TRIM2 could aggravate cell proliferation, invasion, and migration in lung cancer by regulating Snail1 ubiquitylation degradation. Our results could provide detailed information for further studies in lung adenocarcinoma.

## Supplementary information

**Additional file 1: Figure S1.** qRT-PCR analysis of EMT and stem cell related biomarkers (A) and (B) qRT-PCR analysis of EMT biomarkers in TRIM2 knockdown treated A549 cells and TRIM2 overexpression treated H322 cells that had been treated with Snail1 knockdown and overexpression treatments. (C) and (D) RT-PCR analysis of stem cell biomarkers in TRIM2 knockdown treated A549 cells and TRIM2 overexpression treated H322 cells that had been treated with Snail1 knockdown and overexpression treatments.

## Data Availability

The datasets used and/or analysed during the current study are available from the corresponding author on reasonable request.

## Abbreviations

NSCLC: Non-small-cell cell lung cancer
SCLC: Small cell lung cancer
TRIM: Tripartite motif protein
TRIM2: Tripartite motif-containing protein 2
NF-L: Neurofilament lightsubstances
Bim: Bcl-2 interminding medial deafeat
SuperTRIgent: SuperPure Total RNA Extraction Reagent
CQ: Chloroquine
Co-IP: Co-immunoprecipitation
CHX: Cycloheximide
VHL: Vonhippel-Lindau
HIF-1α: Hypoxia-inducible factors-1α
HIF-2α: Hypoxia-inducible factors-2α
