# Supplementary figures and images for: TRIM2 directly deubiquitinates and stabilizes Snail1 protein, mediating proliferation and metastasis of lung adenocarcinoma
Source: Cancer Cell Int. 2020 Jun 10;20:228. doi: 10.1186/s12935-020-01316-6 (PMC7288537; doi:10.1186/s12935-020-01316-6)

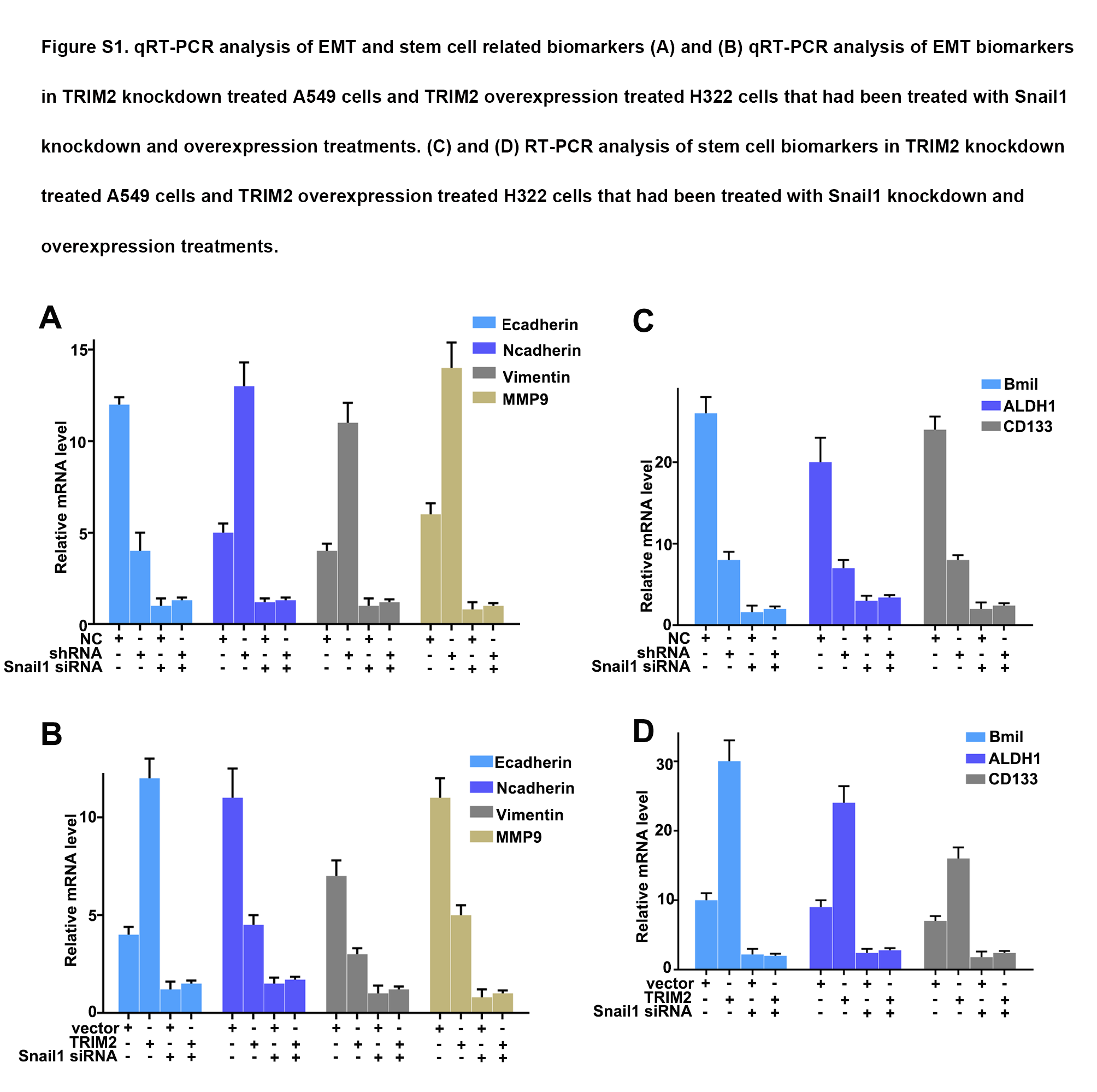

Supplement: Supplementary file 1 — Additional file 1: Figure S1. qRT-PCR analysis of EMT and stem cell related biomarkers (A) and (B) qRT-PCR analysis of EMT biomarkers in TRIM2 knockdown treated A549 cells and TRIM2 overexpression treated H322 cells that had been treated with Snail1 knockdown and overexpression treatments. (C) and (D) RT-PCR analysis of stem cell biomarkers in TRIM2 knockdown treated A549 cells and TRIM2 overexpression treated H322 cells that had been treated with Snail1 knockdown and overexpression treatments. [file 12935_2020_1316_MOESM1_ESM.tif]
